# Selective targeting of liver cancer with the endothelial marker CD146

**DOI:** 10.18632/oncotarget.2345

**Published:** 2014-08-13

**Authors:** Stefan Thomann, Thomas Longerich, Alexandr V. Bazhin, Walter Mier, Peter Schemmer, Eduard Ryschich

**Affiliations:** ^1^ Department of Surgery, University of Heidelberg, Germany; ^2^ Department of Pathology, University of Heidelberg, Germany; ^3^ Department of Nuclear Medicine, University of Heidelberg, Germany

**Keywords:** hepatocellular cancer, drug delivery, tumor endothelium

## Abstract

Hepatocellular carcinomas are well-vascularized tumors; the endothelial cells in these tumors have a specific phenotype. Our aim was to develop a new approach for tumor-specific drug delivery with monoclonal antibody targeting of endothelial ligands. CD146, a molecule expressed on the endothelial surface of hepatocellular carcinoma, was identified as a promising candidate for targeting. In the present study, endothelial cells immediately captured circulating anti-CD146 (ME-9F1) antibody, while antibody binding in tumors was significantly higher than in hepatic endothelium. Macroscopically, after intravenous injection, there were no differences in the mean accumulation of anti-CD146 antibody in tumor compared to liver tissue, due to a compensating higher blood vessel density in the liver tissue. Additional blockade of nontumoral epitopes and intra-arterial administration, improved selective antibody capture in the tumor microvasculature and largely prevented antibody distribution in the lung and liver. The potential practical use of this approach was demonstrated by imaging of radionuclide-labeled ME-9F1 antibody, which showed excellent tumor-selective uptake. Our results provide a promising principle for the use of endothelial markers for intratumoral drug delivery. Tumor endothelium–based access might offer new opportunities for the imaging and therapy of hepatocellular carcinoma and other liver malignancies.

## INTRODUCTION

The growth of hepatocellular carcinoma (HCC) strongly depends on the continuous development of blood vessels to form a tumor vascular system [1]. The tumor vasculature connects the tumor with the host and supplies the tumor with oxygen and nutrients. Further, it drains away metabolic and other tumor products [2]. Because of its important biological role in tumor progression, the tumor vascular system represents a potential diagnostic and therapeutic target [3]. There are two types of vascular-based strategies. Anti-angiogenic therapy is directed against the formation of new tumor blood vessels and has been established as the therapy of choice against several solid tumors [4]. Vascular-targeted therapy includes different strategies that do not directly attack tumor blood vessels; instead, the tumor vascular system is used for imaging and tumor-specific drug delivery [3].

The phenotype of endothelial cells in HCC differs from the cell-surface expression profile of hepatic endothelial cells [5;6]. One of the molecules overexpressed on tumor endothelial cells (TECs) is CD146 [7], a cell-adhesion molecule of the immunoglobulin superfamily [8]. The functions of CD146 remain relatively unexplored. CD146 is known to participate in angiogenesis [9] and to promote leukocyte adhesion to endothelium [10]. In the present study, we demonstrated that CD146 is overexpressed on TECs in mouse tumors and in a fraction of human HCCs. The expression of CD146 on tumor endothelium was utilized in a new approach for improved HCC-specific drug delivery and could potentially be applied to the imaging and therapy of liver tumors.

## RESULTS

### CD146 overexpressed in tumor blood vessels in mouse and human HCC

**Figure 1:**
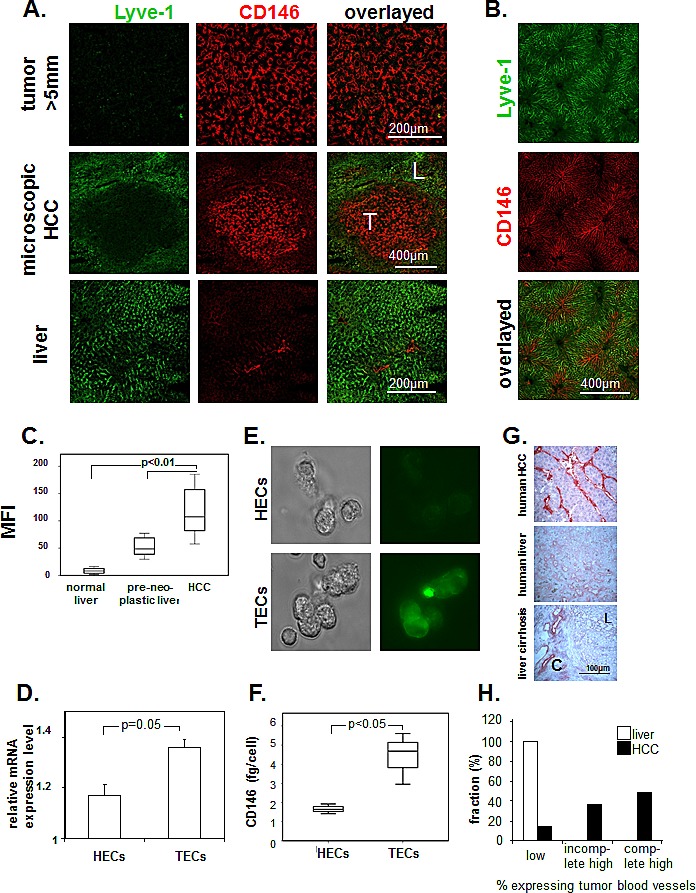
Expression of CD146 on endothelium in murine and human hepatocellular carcinoma (A, B) Representative images of immunofluorescence labeling with Alexa Fluor 488–conjugated anti-Lyve-1 (green) and PE-conjugated anti-CD146 antibodies (red). LSCM of histological slides (A) and whole-mount tissue after intravenous injection (B). Lyve-1 was strongly expressed by normal sinusoidal endothelial cells. High levels of CD146 staining were found in all tumor blood vessels, in microvessels of the periportal area, and in zone 1 of the acinus in the liver. T, tumor; L, liver. (C) Image-based analysis of CD146 staining on histological slides. We observed a high mean fluorescence intensity of CD146 on tumor blood vessels. (D–F) Comparison of CD146 expression in isolated HECs and TECs. mRNA levels (D), representative fluorescence staining with Alexa Fluor 488–ME-9F1 mAb (E), and ELISA of cell lysates (F). CD146 expression was significantly higher in TECs compared to HECs (*P*<0.05). (G, H) Expression of CD146 on endothelium in human tissue; immunohistochemical staining of CD146. (G): Representative images of snap-frozen tissue, L, liver tissue, C, connective tissue. (H): Sample distribution according to expression intensity, formalin-fixed samples of 41 tumors and 3 livers were included into the analysis. CD146 was overexpressed in the majority of human HCC samples.

CD146 expression was studied by immunofluorescence of mouse tissue with ME-9F1 mAb. CD146 was homogenously expressed on all tumor blood vessels (Fig. 1A). The overexpression was independent of tumor size and was observed even in microscopic tumors (200–500 μm). Hepatic sinusoidal blood vessels showed a low reactivity to anti-CD146 mAb, but Lyve-1 was highly expressed (Fig. 1A, B). In liver tissue, high expression of CD146 was mainly present in Lyve-1–negative blood vessels in periportal fields and in zone 1 of the liver acinus (Fig. 1A, B). Quantitative analysis using fluorescence-based imaging demonstrated a significantly higher level of CD146 in tumor blood vessels compared to healthy and preneoplastic liver tissue from AlbTag mice (Fig. 1C). Double staining of Lyve-1/CD146 allowed us to distinguish between Lyve-1(-)CD146^high^ tumor blood vessels in HCC and Lyve-1(+)CD146^low^ tissue in peritumoral liver (Fig. 1A).

Gene expression levels and the exact concentration of CD146 protein were measured in isolated hepatic and tumor endothelial cells. Both gene expression (Fig. 1D) and CD146 protein concentration (Fig. 1E, F) were significantly higher in TECs compared to HECs (*P*=0.05).

CD146 expression was also studied in formalin-fixed and frozen human HCC and normal liver specimens. Immunohistological staining of frozen tissue revealed the presence of CD146^high^ and CD146^low^ blood vessels (Fig. 1D), whereas only CD146^high^ blood vessels were stained in formalin-fixed tissue. Identical to what we observed in mouse tissue, human sinusoidal blood vessels showed low CD146 expression levels. Endothelial expression of CD146 in human HCCs was heterogeneous: the majority of tumor samples showed complete (49%) or incomplete (37%) high expression, whereas 15% of samples had low expression of CD146 (Fig. 1G, H). Cirrhosis did not change CD146 expression in sinusoidal blood vessels, but fibrotic connective tissue shows CD146-positive blood vessels (Fig. 1G). There was no relationship between differentiation grade and CD146 expression (Table 1).

**Table 1:** Number of tumor samples depending on CD146 expression and differentiation grade (G)

G grade	Number of tumor samples		
	CD146 expression		
	Low/absent	incomplete high	complete high
1		3	
2	4	3	12
3	2	9	7
4			1
total	6	15	20

### Endothelial cells immediately capture circulating ME-9F1 mAb

**Figure 2:**
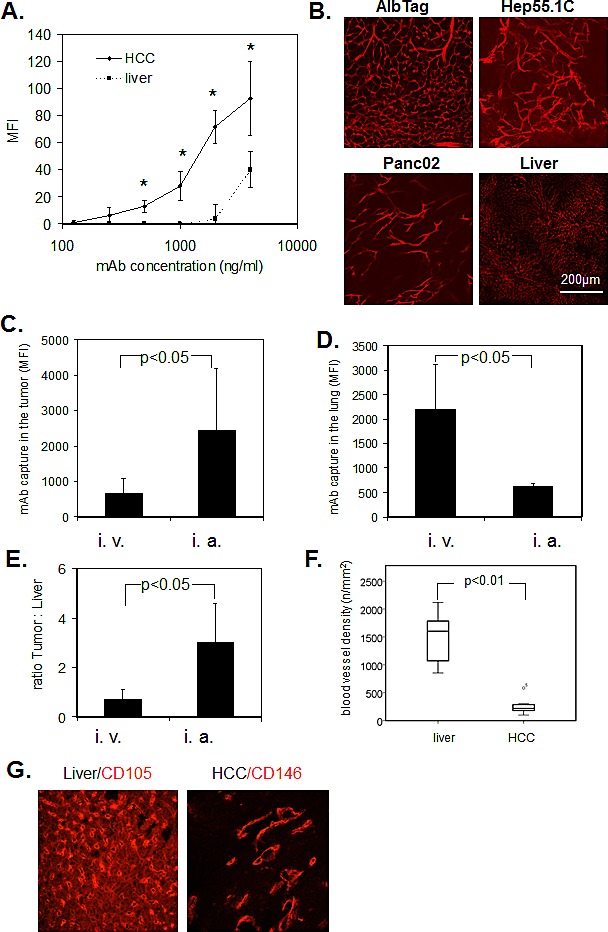
Immediate binding of ME-9F1 mAb to endothelial cells (A) Image-based immunofluorescence analysis of mAb binding to histological slides after 5 s incubation. ME-9F1 showed immediate concentration-dependent binding to tumor endothelial cells, which was significantly higher than binding to hepatic endothelial cells (*P<*0.05). *Indicates significant differences between tumor and liver tissue. (B) Images of PE-conjugated ME-9F1 mAb binding to tumor endothelial cells *in vivo*; laser scanning confocal microscopy. Intravenous injection of mAb resulted in excellent visualization of the tumor vascular system in different mouse tumor models. (C–E) Capture of ME-9F1 mAb in tumor and lung tissue after intravenous (i.v.) and intra-arterial (i.a.) injection. mAb binding in tumor (C) and tumor:liver ratio (E) after intra-arterial application was significantly higher than after intravenous injection. Higher mAb binding in the lung was found after intravenous injection (D). (F–G) Blood vessel density (F) and representative images of blood vessel staining in HCC and liver tissue using anti-CD146 or anti-CD105 mAb (G). Tumor blood vessel density in the liver was significantly higher than in HCC from AlbTag mice (*P*<0.05).

To study ME-9F1 affinity *ex vivo*, we analyzed the CD146-specific MFI in histological sections, which depended upon the addition of different mAb concentrations and a subsequent standardized incubation time. A 5 s exposure to 2–4 μg/ml PE-conjugated ME-9F1 mAb was sufficient to achieve substantial staining of tumor blood vessels, whereas staining of blood vessels in the liver was significantly lower (Fig. 2A). Furthermore, intravenous injection of PE-labeled ME-9F1 mAb immediately stained tumor blood vessels in AlbTag, Hep55.1C, and Panc02 tumor models *in vivo* (Fig. 2B). The epitope for ME-9F1 mAb was thus accesible on the intraluminal surface of endothelium.

Fluorimetric analysis of tissue homogenates showed that the concentration of captured mAb in HCC, as well as the “tumor:liver” ratio, were significantly higher after intra-arterial compared to intravenous injection (Fig. 2C, E). In contrast to tumor tissue, the mean mAb fluorescence in lung tissue was significantly higher after intravenous compared to intra-arterial mAb application (Fig. 2D).

### Negative impact of hepatic blood vessel density on tumor:liver ratio of captured ME-9F1 mAb

**Figure 3:**
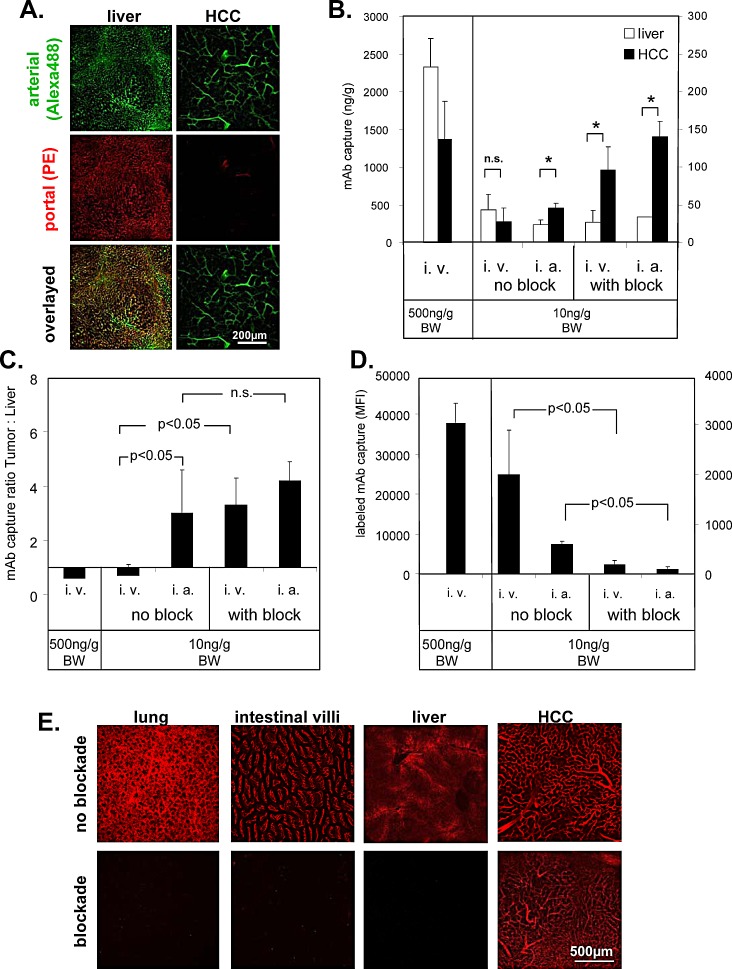
Selective access to tumor vasculature using PE-conjugated ME-9F1 mAb (A) Representative LSCM images of selective arterial blood supply to HCC. Alexa Fluor 488 (green)- or PE (red)-labeled ME-9F1 mAb were injected during alternate clamping of the hepatic artery or portal vein. Blood vessels in the liver, as well as in small tumors, were labeled with both Alexa Fluor 488 and PE (mixed arterial and portal blood supply). Microvessels in larger tumors were labeled mainly with PE-conjugated mAb (selective arterial blood supply). (B–D) Selective enrichment of labeled ME-9F1 mAb using bioavailability blockade of nontumoral epitopes or/and intra-arterial injection. Diagrams show mAb content in the tumor, liver (C), and lung (E) and the mAb tumor:liver ratio (D). Bioavailability blockade of nontumoral epitopes by unconjugated mAb and intra-arterial injection of 10 ng/g BW mAb improved selective accumulation of labeled mAb in tumor tissue and strongly reduced mAb load in the lung. *Indicates significant differences between tumor and liver tissue. (E) LSCM images of different organs without and with blockade of nontumoral epitopes. Blockade of nontumoral epitopes resulted in visualization of solid tumors (>5mm) through selective labeling of tumor vasculature, whereas the fluorescence signal in the liver and other organs was strongly inhibited after blockade.

The mean blood vessel density in the liver was significantly higher than in tumor tissue (Fig. 3F, G). Although binding of ME-9F1 mAb on TECs was significantly higher than in HECs (Fig. 1E,F), the mAb content per gram of tissue was not significantly different between liver and HCC tissue after intravenous injection (Fig. 3B). Furthermore, PE-labeled ME-9F1 mAb bound to endothelial cells in other tissues, such as lung, intestinal villi, and pancreas (Fig. 3E). No binding of isotype mAb to endothelium was found.

### Hepatic artery clamping interrupts microperfusion in the tumor but not in the liver

To study blood vessels with differential connections to the arterial and portal venous blood supply, Alexa Fluor 488– or PE-labeled ME-9F1 mAbs were injected during alternate clamping of either the hepatic artery or portal vein. mAb binding was analyzed by LSCM. Almost all blood vessels in the liver, as well as in small tumors (<5 mm) were labeled by both Alexa Fluor 488 and PE, demonstrating their connection to both arterial and portal blood supply. All microvessels in larger tumors (>5 mm) were labeled by Alexa Fluor 488; only a few blood vessels were labeled by PE-mAb. This demonstrates that tumor microvessels in larger tumors were perfused only when the hepatic artery was not clamped (Fig. 3A).

### Blocked bioavailability of nontumoral epitopes or/and intra-arterial application of ME-9F1 mAb provides selective access to the tumor

Intravenously injected PE-labeled ME-9F1 bound to endothelium in liver and tumor tissue, but the mean surface-bound mAb concentrations were not significantly different (Fig. 3B). The capture of mAb due to endothelium binding resulted in rapid mAb clearance from the circulation within several minutes if the injected mAb dose did not exceed 500 ng/g BW (Supplementary Fig. 1A). Injection of mAb in doses of >500 ng/g BW exceeded the endothelial capacity to clear the antibody from the blood and resulted in high concentrations of circulating mAb (Supplementary Fig. 1A).

Application of unlabeled ME-9F1 mAb occupied free CD146 epitopes on endothelium and prevented binding of the subsequently injected fluorescent mAb in the liver (Fig. 3B, E) and lung (Fig. 3D). Temporary clamping of the hepatic artery prevented occupation of tumor endothelial epitopes by unconjugated mAb and improved selective binding of conjugated mAb to tumor endothelium after removal of the clamp and subsequent injection of fluorescence labeled ME-9F1(Fig. 3B, E).

Direct mAb injection into the hepatic artery significantly increased the capture of mAb in the tumor (Fig. 3B) and significantly enhanced the tumor:liver ratio (Fig. 3C). The combination of blockade and intra-arterial mAb injection did not result in further significant increases in intratumoral mAb binding (Fig. 3C). mAb concentrations in the liver were increased 5 h after intravenous injection (Supplementary Fig. 1B). This was accompanied by high nonvascular fluorescence of liver tissue and led to strong attenuation of the tumor:liver ratio of fluorescent mAb (Supplementary Fig. 1B).

### Intraarterial hepatic perfusion and tumor imaging with 125I-conjugated ME-9F1 mAb

**Figure 4:**
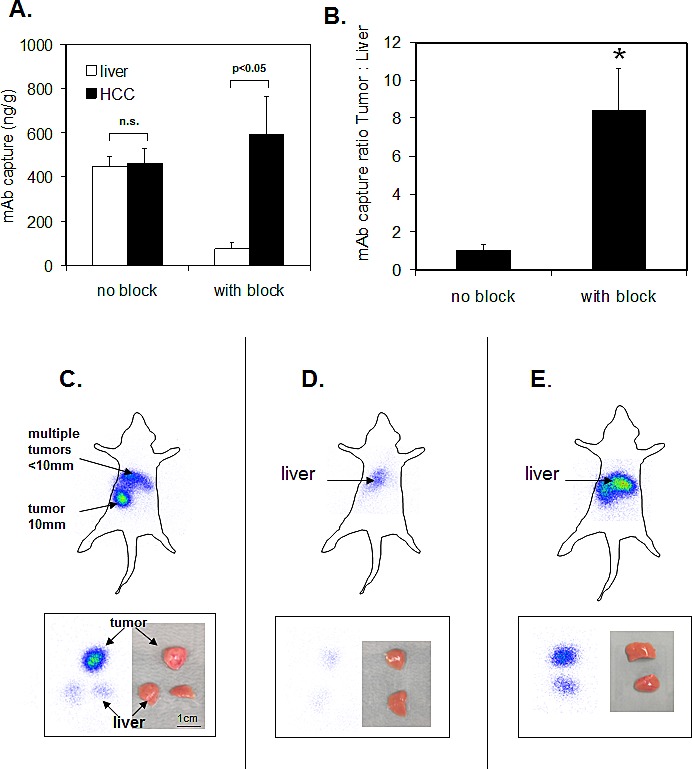
Intraarterial hepatic perfusion and static planar imaging of the whole body (top panels) and tissue pieces (bottom panels) (A-B) The combination of a reduction in the nontumoral epitope bioavailability and administration of PE-conjugated ME-9F1 into the hepatic artery led to preferential accumulation of labeled mAb in tumor vessels and resulted in the high tumor:liver ratio. *p<0.05. (C) Combination of bioavailability blockade of nontumoral epitopes and intra-arterial perfusion with I^125^-conjugated ME-9F1 mAb in a tumor-bearing mouse. Multiple small HCCs and one large tumor were macroscopically identified in the liver. Tumor tissue produced a strong signal, whereas a very weak signal was detected in the liver. T, tumor; L, liver. (D) Combination of nontumoral epitope blockade and intra-arterial perfusion with I^125^-conjugated ME-9F1 mAb in a tumor-free mouse. Weak signal in the liver was detected. (E) Intraportal perfusion with I^125^-conjugated ME-9F1 mAb without blockade in a tumor-free mouse. Strong signal in the liver tissue was detected.

The combination of a reduction in the nontumoral epitope bioavailability and administration of PE-conjugated ME-9F1 into the hepatic artery resulted in preferential accumulation of labeled mAb in tumor vessels (Fig. 4A-B). The mean tumor:liver ratio was increased from 1.1 without block to 8.4 after blocking approach (Fig. 4B).

The application of ^125^I -conjugated mAb tumors showed a high radioactivie signal after application of I^125^-conjugated ME-9F1 mAb and block of nontumoral epitope bioavailability. Peritumoral liver vessels emitted a very weak signal (Fig. 4C). Only low emission was measured in tumor-free liver tissue after the same procedure (Fig. 4D). Intraportal injection of ^125^I-conjugated ME-9F1 mAb without pretreatment produced a strong scintigraphic signal in the liver (Fig. 4E).

## DISCUSSION

The present investigation validated a new approach to access liver tumors. This approach utilizes the binding of specific ligands, such as antibodies, to markers expressed on the intraluminal surface of tumor endothelium. CD146 was approximately 2.6-fold overexpressed on mouse tumor endothelial cells compared to liver endothelial cells. The lower difference (1.2-fold) at gene expression level can be explained by posttranslational regulation of protein synthesis. This high overexpression of CD146 in tumor endothelium occurred in two HCC models and in mouse pancreatic cancer inoculated in the liver. Our findings regarding CD146 overexpression in mouse models are likely to be relevant to humans, since the majority of human HCCs overexpress CD146 on endothelium. Indeed, CD146 represents only one potential molecule for use in further translational studies. Other endothelial markers with higher tumor specificity may be identified. So-called “tumor endothelial markers” (TEMs) could represent alternatives to CD146 [7;11]. However, the association between tumors and TEMs is relative, since TEMs are not restricted to tumor tissue and can also be found in different organs and cell types [12]. Furthermore, TEMs may be expressed only in a fraction of tumor blood vessels [11] and may not provide the homogeneity required by the present approach.

The present study also demonstrated that endothelium-directed mAbs such as ME-9F1 bound immediately to their epitope; this resulted in excellent labeling of tumor blood vessels at the microscopic level. Interestingly, the high tumor:liver ratio of anti-CD146 mAb binding at the level of single endothelial cells disappeared at the macroscopic level. This can be explained by the higher blood vessel density in the liver, which increased the density of mAb binding in the liver at the macroscopic level and decreased the microscopic contrast of mAb bound to tumor endothelium.

mAb capture in the lung after intravenous administration was higher than after intra-arterial injection, whereas the opposite relationship was found in tumor tissue. This difference was due to the elimination of mAb during the first passage through the next vascularized organ. This organ is the lung after intravenous injection and the tumor after intra-arterial injection. The mAb captured in the successive organ may result in decreased concentrations of mAb reaching subsequent organs. In this case, mAb capture in the lung and in the tumor after intravenous and intra-arterial administration, respectively, follows the principle in which concentrations of the drug at the site of intake is higher than its concentration at the site of outcome. In pharmacokinetics, this principle is commonly known as the “first-pass effect,” and it has been described for the intrahepatic capture and elimination of several drugs, such as opioids and nitroglycerin [13]. In the current study, intravenous administration also led to reduced mAb bioavailability in the effector organ, not through enzymatic metabolism, but through direct epitope binding to the vasculature. Taking this principle into consideration will help to decrease the drug load in lung tissue and enhance tumoral drug bioavailability by directly injecting the drug into the tumor-feeding artery.

In the present study, we used anti-CD146 immunoglobulin G (IgG). Other molecule types, such as IgG fragments, natural soluble receptors, and peptides with high binding affinity to the intraluminal endothelial surface represent promising alternatives to conventional IgG. For example, there are two natural ligands/receptors to CD146 that could be investigated as alternatives to mAb: laminin-411 [14] and VEGFR2 [15]. Their functionality should be evaluated in further studies.

As mentioned above, CD146 is a ubiquitous endothelial marker that is expressed in other organotypic endothelial cells. Therefore, high binding of PE-conjugated ME-9F1 mAb in the lung, intestinal villi, and pancreas was detected after systemic mAb administration. The liver vasculature is connected to both the portal vein and hepatic artery, whereas the artery usually supplies HCCs [16]. As shown in the present study, temporary clamping of the hepatic artery only interrupted blood supply in the tumor microvasculature, demonstrating the exclusive arterial blood supply of the tumor. Furthermore, to achieve selective mAb binding in the tumor, the labeled mAb was injected into the hepatic artery; alternatively, the bioavailability of nontumoral epitopes was blocked prior to injection of the targeting mAb. Both tools may be relevant for translation to a clinical setting. Arterial access to the HCC is routinely performed in transarterial chemo- or radioembolization [17;18] and in continuous transarterial chemotherapy [19]. Temporal clamping of the hepatic artery is a familiar practice and is widely used in Pringle's maneuver (portal triad clamping) during liver surgery [20]. The blocking procedure does not limit the potential widespread use of the antibody. The dose of blocking antibody is only 500ng/g BW (=0.5mg/kg) in the case of anti-CD146. This dose is substantially lower than the dose of established therapeutic antibodies. For example, the single dose of antibodies such as anti-VEGF-A [21;22], anti-HER2 [21] or “checkpoint blocking” antibodies [23] can reach 15, 8 or 10mg/kg, respectively.

The results of the present study show that the intrahepatic mAb accumulation had increased several hours after injection; this indicates that transhepatic mAb metabolization was occurring, which had a negative impact on the tumor:liver ratio. The use of alternative substances or drug-bearing nanocarriers that bind to tumor vasculature but are not metabolized in the liver would prevent the later intrahepatic drug accumulation.

The novel targeting approach described herein has high clinical relevance, but it requires further development before it can be translated to therapeutic strategies. The present study demonstrated that the use of radionuclide-conjugated mAbs allows tumors to be imaged by clinically established techniques. Administration of ^125^I-mAb into the liver isolated from the circulatory system corresponds to the basic principle used in hepatic vascular exclusion or chemosaturation of liver tumors [24;25]. The high-quality scintigraphic imaging that accompanied the use of radionuclide-labeled mAbs supports the use of this technique for tumor-specific drug delivery and provides a rationale for the proposed approach.

The approach can also represent a very promising tool for tumor therapy. However, therapeutic evaluation is very difficult in murine models, since repeated surgery on hepatic artery (clamping, arterial injection) would be required. Intra-arterial application of carriers based on tumor–endothelium targeting for imaging and therapy is the focus of ongoing experiments.

In summary, the present study describes a new approach for tumor specific drug delivery in HCC and liver tumors. This approach utilizes the binding of specific ligand to endothelial markers expressed on the intraluminal surface of tumor endothelium. In the present study, its technical feasibility was examplified using monoclonal antibody binding to endothelial marker CD146. The tumor-specific bioavailability of CD146 can be substantially increased by use of additional methods such as blockade of nontumoral epitopes and by intraarterial application.

## METHODS

### Mouse tumor models

AlbTag mice expressing the oncogene SV40 large T antigen under the control of the albumin promoter were used at the stage of spontaneous HCC development [26]. Hep55.1C (HCC) or Panc02 (pancreatic cancer) cells (5×10^4^ cells) were inoculated into the livers of transplantable model mice (C57/Bl6). The tumor study was performed 18–20 d after inoculation. All animal experiments were approved by the local committee for animal care.

### Immunohistochemistry and immunofluorescence

Human tissue samples were provided by the tissue bank of the National Center for Tumor Diseases (NCT, Heidelberg, Germany) in accordance with the regulations of the tissue bank. and the approval of the ethics committee of the University of Heidelberg. Snap-frozen samples of eight HCCs, three normal and five cirrhotic livers as well as formalin-fixed samples of 41 HCCs and three livers were used.

The following monoclonal antibodies (mAbs) were used: unconjugated phycoerythrin (PE)- or Alexa Fluor 488–conjugated anti-mouse CD146 (ME-9F1), PE-conjugated anti-CD105 (FIT-22), Alexa Fluor 488–conjugated Lyve-1 (ALY7), and Alexa Fluor 488–conjugated anti-human CD146 (SHM-57) (all mAbs from Biolegend, San Diego, CA, USA). Anti-human CD146 mAb was purchased from Epitomics (Burlingame, CA, USA). Tissue slides (7 μm thickness) were stained by direct immunofluorescence or indirect three-step immunohistochemistry with the LSAB kit (Dako, Carpinteria, CA, USA) and counterstained with Mayer's hemalaun (Fluka, Steinheim, Germany).

### Image-based quantitative analysis of immunofluorescence staining and blood vessel density

Bound fluorescent mAb was visualized with fluorescence microscopy (Observer.Z1; Zeiss, Jena, Germany). The mean integrated density at three areas per power field containing at least five vessels was measured by immunofluorescence imaging and ImageJ software (*National Institutes of Health*, Bethesda, Maryland, USA) [27]. Each value was corrected for background and expressed as mean fluorescence intensity (MFI). Tumor tissue, peritumoral liver (<500 μm distance from tumor) and normal liver were analyzed (*n*=6 for each).

To calculate blood vessel density, tumor or liver slides were stained with anti-CD146 or anti-CD105, respectively (*n*=11). The number of blood vessels was counted with Histo Software (Dr. Groβ, University of Heidelberg) and expressed per mm^2^.

To stain endothelial cells *in vivo*, PE-conjugated ani-CD146 (50 ng/g body weight [BW]) and anti-Lyve-1 (200 ng/g BW) mAbs were injected intravenously in tumor-free (*n*=2) or tumor-bearing mice. The tissue was dissected 15 min after injection and analyzed with the Nikon A1Rsi confocal laser scanning system (LSCM; Nikon Europe, Dusseldorf, Germany) as whole-mount tissue.

### Endothelial cell isolation, qRT-PCR, and ELISA

Tumor-bearing AlbTag or normal C3Heb/F mice (11–12 weeks old) were used. Hepatic endothelial cells (HECs) or tumor endothelial cells (TECs) were isolated by collagenase digestion and magnetic separation with anti-CD31–coated magnetic beads (Miltenyi Biotec, Bergisch Gladbach, Germany), as previously described [5]. Isolated cells were stained with Alexa Fluor 488–conjugated ME-9F1.

For real-time reverse transcription PCR (qRT-PCR), total RNA from endothelial cells was isolated with the RNeasy mini kit (Qiagen, Hilden, Germany) according to the manufacturer's instructions. qRT-PCR analysis was performed with the QuantiFast SYBR Green RT-PCR kit and QuantiTect Primer (Qiagen). Standardization of samples was achieved by dividing the C_t_ of the target gene by that of the endogenous reference genes β-actin and GAPDH (Qiagen). For each experiment, melting-curve analysis and gel electrophoresis of PCR products were performed to exclude primer dimers. Data were analyzed by the comparative C_t_ method.

For ELISA, lysates from isolated endothelial cells were used. CD146 protein concentration was determined with the mouse MCAM-ELISA kit (USCN, Wuhan, China) according to the manufacturer's instructions.

### Fluorimetry of tissue homogenates

Mice were anesthetized with 40 mg/kg ketamine (Pfizer, Berlin, Germany) and 10 mg/kg xylazine (Bayer, Leverkusen, Germany). Antibodies were injected through the jugular vein or into the hepatic artery. To access the hepatic artery, a 34G needle (Hamilton, Bonaduz, Switzerland) was inserted into the superior pancreaticoduodenal artery and moved forward into the hepatic artery. To prevent blood loss from the puncture site, the needle was fixed in the pancreaticoduodenal artery with 10.0 thread (Covidien, Mansfield, MA, USA). For temporary discontinuation of hepatic artery blood flow, the hepatic artery was clamped with a microclip (Fine Science Tools, Heidelberg, Germany). To block the bioavailability of nontumoral epitopes, 500 ng/g BW unconjugated ME-9F1 mAb was injected intravenously. Five minutes later, 10 ng/g BW PE-conjugated ME-9F1 mAb (100 μl) was injected intravenously or into the hepatic artery within 5–10 s. The microclip was removed 1 min after injection. Five minutes later, the mouse was sacrificed. Blood from the liver was removed by intraportal perfusion with 5 ml of saline. The tissue sample (liver, tumor, or lung) was dissected, weighed, diluted with phosphate-buffered saline solution (1:1), and homogenized with a manual grinder (neoLab, Heidelberg, Germany). The content of PE-conjugated mAb in the tissue homogenates was determined in 384-well plates (Greiner, Frickenhausen, Germany) in a fluorimeter (BMG Labtech, Ortenberg, Germany). Preliminary analyses showed that PE fluorescence was not affected after dilution in liver and tumor homogenates, but it was strongly reduced in the lung homogenate. Therefore, mAb concentrations in the liver and tumor were calculated in ng/g using a calibration curve, whereas the mAb concentration in lung homogenate was used as a raw MFI value. Three mice per group were used.

To study the differential connection of tumor and liver tissue to arterial or portal venous blood supplies, 500 ng/g BW Alexa Fluor 488–conjugated ME-9F1 mAb was injected intravenously, while the portal vein was clamped. After 5 min, the portal vein perfusion was opened, but the hepatic artery was clamped and PE-labeled ME-9F1 mAb (20 ng/g BW) was injected intravenously. Five minutes later, the tumor and liver tissue were dissected and analyzed by LSCM (Nikon). Microvessels perfused through the hepatic artery were thus labeled with Alexa Fluor 488, whereas blood vessels were labeled with PE, which indicated a connection to the portal vein. PE-conjugated rat IgG2a (cloneRTK2758, Biolegend) was used as isotype control mAb.

### Transarterial hepatic perfusion and scintigraphic imaging

In six animals, bioavailability of non-tumoral epitopes was blocked with 1000ng/g BW for 5min as described above. Animals were sacrificed and 1ml of PE-labeled ME-9F1 mAb solution (4μg/ml) was perfused through the hepatic artery for 2min. Unbound mAb was removed by extensive intraarterial and intraportal perfusion of saline.

ME-9F1 mAb was conjugated with ^125^I, as previously described [28]. Tumor-bearing AlbTag and tumor-free animals were sacrificed. The blocking of nontumoral epitopes was performed by intraportal perfusion with 10 μg of ME-9F1 mAb diluted in 0.5 ml of saline for 15 min. Subsequently, 5 μg of ^125^I-conjugated ME-9F1 mAb diluted in 500 μl of saline was perfused through the hepatic artery for 5 min. Finally, unbound mAb was removed by intraarterial and intraportal perfusion of saline. In tumor-free mice, intraportal perfusion with ^125^I-mAb was performed without pretreatment with ME-9F1 mAb. The animal or the dissected tumor/liver tissue were placed on a gamma imager (Biospace Lab, Paris, France) equipped with a high-energy collimator, and images were recorded over 10 min. Each experiment was performed twice.

### Statistical analysis

Statistical analysis was performed with IBM SPSS software (IBM, New York, NY, USA). Data are shown as mean ± standard deviation (SD). To study differences between the groups, analysis of variance or Mann-Whitney U-test were used, as appropriate. *P*<0.05 was considered significant.

## SUPPLEMENTARY MATERIAL AND FIGURES


