# Supplementary material for: Selective targeting of liver cancer with the endothelial marker CD146
Source: Oncotarget. 2014 Aug 13;5(18):8614–24. doi: 10.18632/oncotarget.2345 (PMC4226708; doi:10.18632/oncotarget.2345)
Supplement: Supplementary file 1 [file oncotarget-05-8614-s001.pdf]

Selective targeting of liver cancer with the endothelial marker CD146

Supplementary Material

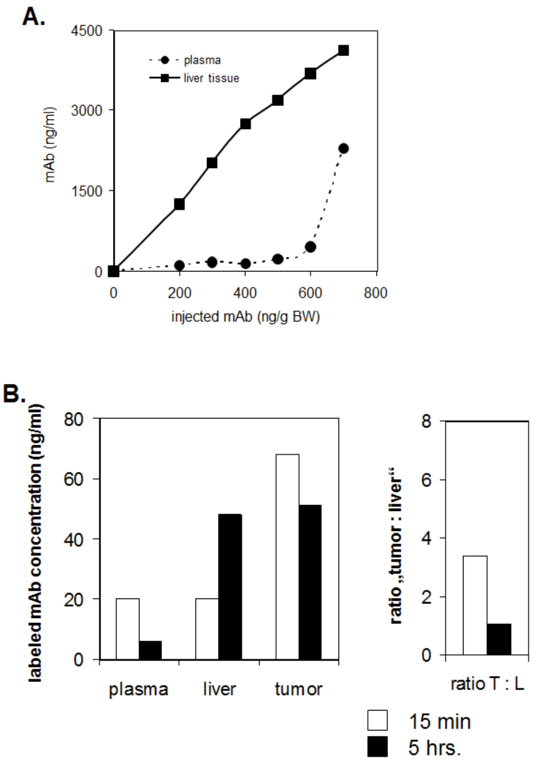

Supplementary Fig. 1: (A) Relationship between dose of injected ME-9F1 mAb and mAb concentration in the blood and liver. Concentration of mAb in liver and plasma of AlbTag mice (10 wk old) was determined 15 min after intravenous injection of different mAb doses. mAb concentrations in the liver showed a positive correlation with the injected dose, whereas mAb concentrations in plasma remained very low up to 500 ng/g BW. Injection of mAb in doses > 500 ng/g BW resulted in increasing concentrations of circulating mAb. **(B) Pharmacodynamics of bound and circulating mAbs.** PE-conjugated ME-9F1 mAb (10 ng/g BW) was injected after blockade of nontumoral epitopes. Intrahepatic and intratumoral concentrations were equalized 5 h after injection.
